# Moderate-to-vigorous intensity physical activity levels of children with intellectual disability during physical education classes

**DOI:** 10.3389/fpubh.2023.1056191

**Published:** 2023-11-10

**Authors:** Yaru Hao, Rizal Razman

**Affiliations:** Faculty of Sports and Exercise Science, Universiti Malaya, Kuala Lumpur, Malaysia

**Keywords:** children, intellectual disability, moderate-to-vigorous intensity physical activity, physical education class, objective measurements

## Abstract

**Background:**

Physical education (PE) class is an excellent way to improve moderate-to-vigorous intensity physical activity (MVPA). Increasing number of research has explored the children’s PA based on movement during PE classes, but data for children with intellectual disability (ID) is still lacking.

**Purpose:**

The purpose of this study was to investigate the current status of MVPA levels of children with ID during PE classes in China, as well as differences of MVPA levels according to gender and grade.

**Methods:**

Accelerometers were used to record MVPA levels of fifty-three children with severe ID from 9 to 16 years of age (mean age: 12.60 ± 1.66 years) during standard PE classes.

**Results:**

The mean time spent in MVPA during PE classes was 8.00 ± 2.10 min, meaning only 22.88% of PE class time was spent in MVPA. As grade levels progresses, time spent in MVPA during PE classes tended to decrease; the fourth-grade children tended to spend more time in MVPA during PE classes compared with the fifth-grade and the sixth-grade (9.15 vs. 7.61 vs. 7.25 min, all *p* < 0.05). Boys spend significantly more time in MVPA during PE classes than girls; both in the entire sample (9.20 vs. 5.70 min) as well as in each grade (9.76 vs. 6.09 min, 9.35 vs. 5.68 min, 8.31 vs. 5.59 min, all *p* < 0.05).

**Conclusion:**

Findings from this study indicate that the proportion of PE class spent in the MVPA of children with ID was lower than the 50% recommended by the U.S. Department of Health and Human Services (DHHS) and U.K. Association for Physical Education (AfPE). And the amount of MVPA participation varied by the grade and gender as well as by the activity performed. Therefore, in order to help children with ID achieve MVPA goals, educators need to reevaluate the PE curriculum as well as take due consideration of grade and gender when devising new content.

## 1. Introduction

Physical activity (PA) is a cornerstone of human health and well-being, with a profound impact on physical and psychological, as well as social aspects of life (1). It is particularly vital for children, shaping their growth and development, fostering cognitive functions, and establishing healthy habits that can endure into adulthood (2, 3). For children with intellectual disability (ID), the importance of maintaining adequate PA levels is magnified. ID encompass a range of conditions characterized by limitations in cognitive functioning and adaptive behavior, affecting an individual’s ability to comprehend, learn, and interact with their environment (4). Thus, children with ID often face unique barriers include motor deficits, communication challenges and sensory sensitivities that may impede their engagement in PA (5, 6). Physical inactivity can lead to a cascade of negative physical and psychological outcomes, further impeding their already complex journey (7). Collins and Staples (8) highlights how reduced PA in children with ID contributes to an increased risk of obesity, cardiovascular diseases, and musculoskeletal disorders. Additionally, physical inactivity can exacerbate the already existing motor deficits, leading to decreased functional independence and reduced overall quality of life (9, 10). The psychological well-being of children with ID is closely intertwined with their PA levels. Lower PA levels has been associated with heightened levels of anxiety, depression, as well as diminished sense of self-worth and confidence in this population (11). Furthermore, a review by Ahn and Fedewa (12) suggests a potential link between inadequate physical activity and cognitive decline in children with ID. Crucially, the gravity of these adverse outcomes is reflected in the significant discrepancy of approximately two decades in life expectancy between children with ID and those without (13).

Increased PA has emerged as a potent avenue for enhancing the physical and psychological health of children with ID. Previous evidence has shown that engaging in regular PA can yield a myriad of positive effects, offering a transformative impact on their overall well-being (14). Physically, heightened PA levels have been associated with improved cardiovascular health, muscular strength, and overall physical functioning among children with ID (15, 16). These benefits are pivotal, as children with ID often face elevated risks of obesity, cardiovascular diseases, and musculoskeletal disorders (17–19). Engaging in moderate-to-vigorous intensity physical activities (MVPA) can help mitigate these risks, promoting better weight management, and fostering healthier cardiovascular and metabolic health biomarkers (20). In addition to the tangible physical gains, increased PA also exerts a profound influence on the psychological health of children with ID. Regular engagement in PA has been linked to reduced levels of anxiety and depression, promoting better emotional well-being (21). Participating in group activities and sports can foster a sense of belonging, social integration, and improved self-esteem among these children (7). The positive interactions and shared experiences inherent in physical activity settings provide opportunities for enhanced socialization, communication skills, and teamwork (22). Moreover, the cognitive benefits of increased PA are noteworthy, regular exercise can lead to improvements in attention, memory, and problem-solving skills, thereby enhancing cognitive functions among children with ID (23, 24). These cognitive enhancements contribute to increased autonomy in daily life. Furthermore, the potential of PA to mitigate the negative effects of sedentary behavior cannot be understated. Children with ID often spend significant time engaged in sedentary activities, which can exacerbate their health challenges and hinder their development (25, 26). By engaging in regular PA, these children can counteract the detrimental impact of sedentary behavior, promoting healthier growth trajectories and better overall health outcomes.

The World Health Organization (WHO) recommends that children with disability aged 5–17 years accumulate at least 1 h of MVPA per day, unfortunately, current data indicates that the percentage of children with ID who meet the recommended levels of PA ranges from only 0 to 42% (27). The PA levels of these children is a matter of concern, as children with ID participate less frequently in PA than their peers without ID (28–30). Many studies have showed that several key factors influence the PA levels of children with ID. Environmental factors play a significant role, including accessibility to appropriate facilities, inclusive physical education (PE) programs, and community resources that promote PA (31, 32). The presence of supportive social networks, such as family members, peers, and PE educators, can positively impact the engagement of children with ID in PA (33–36). Moreover, individual factors such as the child’s age, gender, severity rating of ID, functional abilities, and personal interests also shape their propensity for PA participation (34, 37, 38). According to recent estimates, there are approximately 1.5 million children with ID in China, reflecting a substantial portion of the overall pediatric population (39). In 2020, there are 2,244 special education schools nationwide, with 881,000 students enrolled, or 2.1 times as many as in 2010 (40). To counter physical inactivity issue, the State Council and the Ministry of Education of the People’s Republic of China have identified and developed methods to improve PA levels in children with ID by cultivating regular exercise habits and ensuring that they participate in no less than 1 h of PA per school day (41, 42). However, recent research still paint a bleak picture, describing Chinese mainland children with ID as being extremely inactive (43).

Studies have shown that children with ID are more likely to engage in activities that are less structured, such as free play or unstructured PA (44). The limited opportunities for structured PA contribute to reduced PA (45). Moreover, the combination of financial pressures and parents’ concerns about safety means that fewer children with ID are able to play games in non-school settings (45, 46). PE classes present an opportunity to bridge these gaps, offering structured environments for PA engagement and skill development. Opportunities for children with ID to participate in PA often occur during school, especially during PE classes, as they can accumulate a large percentage of PA during class hours (47). PE class offers a regulated opportunity for usually qualified, responsible teachers to introduce PA and lifestyle skills and knowledge in a structured way to all children, within a safe and supportive environment (48), thereby contributing to the achievement of recommended PA guidelines. Furthermore, the positive experiences and skills gained from PE classes often extend beyond the classroom, influencing children’s choices and behaviors in leisure time and extracurricular activities (49). The role of PE classes becomes increasingly crucial in equipping children with ID with the tools and motivation to lead physically active lives.

MVPA levels of typically developing children during PE classes has been studied extensively. Although there are clear recommendations about MVPA, both the latest systematic reviews and meta-analyses had indicated that MVPA levels during elementary school and secondary school PE classes do not meet the U.S. Department of Health and Human Services (50) and the U.K. Association for Physical Education (51) recommendation of at least 50% of class time being MVPA (52, 53). Studies have shown that programs designed to improve the quality of PE can increase the amount of time that students are engaged in MVPA to more than 50% of PE class time (54–56); this is one of the most critical outcome indicators for determining the quality of PE class. As with many countries, in China, PA levels during PE classes is also a cause for concern, with MVPA amounting to between only 10.4 and 57.4% of class time (57–60). In addition, some studies (61, 62) have noted differences between boys and girls, as well as differences between different grade levels in PA during PE classes. In general, boys spent a greater proportion of class time engaged in MVPA than girls, and older grades spent more proportion of class time engaged in MVPA than those in younger grades.

Despite the growing body of research on PA in children with ID, there remains a notable gap in the understanding of the specific PA levels of children with ID during PE classes, particularly within the Chinese context. To our knowledge, there are only two studies that focused on PA levels during PE classes in children with ID in Hong Kong and Taiwan (63, 64). They used System for Observing Fitness Instruction Time (SOFIT) and Actigraph GT1M accelerometer, respectively, to investigate PA levels and the results showed that participants failed to achieve the recommended intensity of PA during PE classes. Even though these studies give us an idea of the PA levels of children with ID during PE classes, important information is lacking; namely MVPA levels during PE classes were not reported for children with ID on China’s mainland, and literature search has not yielded data on investigating possible gender and grade differences in MVPA among children with ID during PE classes - making it difficult to develop specific strategies to increase PA levels.

Therefore, the current study seeks to address this gap by (1) investigating the percentage of class time that children with ID engage in MVPA during PE classes; as well as (2) to investigating differences of the time spent in MVPA during PE class according to gender and grade. The findings of this study are essential for designing targeted interventions that optimize the PA experiences of children with ID, potentially leading to more effective and personalized PE curricula. Furthermore, the investigation of this phenomenon within the Chinese educational context adds a unique dimension to the global discourse on inclusive PE.

## 2. Methods

### 2.1. Participants

This cross-sectional study was conducted in April 2022. Fifty-eight students were initially recruited to participate in the study, however, the data from five students (8.62%) had to be excluded due to technical issues with the accelerometer data for one of the days, along with instances of illness-related issues. Eventually, data on fifty-three children (35 boys and 18 girls) with severe ID, aged 9–16 years (mean age: 12.60 ± 1.66), grades four through six (18 in fourth-grade, 17 in fifth-grade, and 18 in sixth-grade), from two public special education schools in a city in eastern China were included in the analysis. All participants included in the study were identified with ID using the Wechsler Intelligence Scale for Children (WISC)-IV, administered by skilled medical professionals in public hospitals during their early childhood. Although the sample sizes for girls was significantly smaller than the sample sizes for boys in this study, previous studies on this type of groups have also used gender-homogeneous sample sizes (63–65). To mitigate potential confounding variables, the recruitment of children with ID adhered to three specific inclusion criteria: (1) the severity ratings of ID was classified as severe [intelligence quotient (IQ) of 25–39], following the severity classification from the Second National Sampling Survey on Disability (66); (2) absence of orthopedic impairment and the capability to engage in physical activity without assistance; (3) and participate in general PE classes in school 100% of the time during the data collection days. Additionally, an exclusion criterion encompassed students with simultaneous autism, cerebral palsy, sensory disabilities, and those with physical impairments. Before starting data collection, permission to conduct the study was obtained from principals and PE teachers at each special education school, and all parents or legal guardians gave their written informed consent. The study was approved by the Universiti Malaya Research Ethics Committee (UMREC) (UM.TNC2/UMREC_1590).

### 2.2. Measurement

#### 2.2.1. MVPA assessment

ActiGraph accelerometer (wGT3X-BT model, ActiGraph, Ft. Walton Beach, United States) was used to assess ID children’s PA during PE classes. It is an objective device for measuring PA and is extensively used in children, including those with ID, and has been demonstrated to be reliable (67). The accelerometer is a small (4.6 cm × 3.3 cm × 1.5 cm) and lightweight (19 g) triaxial device that measures and records dynamic range +/− 8 g and has a sample rate ranging from 30 to 100 Hz.

For this study, the device sampling rate was set at 30 Hz. Data was collected in 10 s epoch, which has been used in previous studies assessing PA levels of children with ID (63, 68). Additionally, children with ID often exhibit a wide range of movement patterns that may include sporadic, involuntary, or non-purposeful movements (69); shorter epoch lengths (e.g., 5 s or less), are more likely to capture these minor, non-relevant movements that do not contribute to the assessment of their PA levels. When assessing PA levels, it’s crucial to differentiate between meaningful activities and trivial movements. Using a 10 s epoch allows for a more comprehensive view of a child’s activity during that time frame. It provides a longer window to assess whether the observed movements are part of a purposeful, sustained activity or simply momentary and inconsequential. Consequently, the 10 s epoch is chosen to effectively capture these fluctuations in their activity and better align with their unique patterns. And the raw output was expressed as activity counts per minute (CPM), which was used to determine the intensity of PA. The original counts were quantified as MVPA (≥2,296 counts per minute) based on the cut-off points suggested by Evenson et al. (70). Evenson cut-off points have been used and validated previously with Chinese children with disabilities (71). We were specifically interested in determining the percentage of time students spent in MVPA during their PE classes. The percentage time in MVPA was summed, and means were calculated to produce MVPA data.

#### 2.2.2. Anthropometry

The participants’ height and weight were obtained from an ultrasonic height and weight integrated machine (model SH-200, SHANGHE, China), and the body mass index (BMI) was calculated as weight in kilograms divided by the square of standing height in meters. Descriptive statistics of the participants included in the final analyses are provided in Table 1, there was no significant difference in BMI between boys and girls (*p* > 0.05).

**Table 1:** Physical characteristics of participants (Mean ± SD).

	Boys (*n* = 35)	Girls (*n* = 18)	*F*	*p*
Age (years)	12.34 ± 1.64	13.11 ± 1.61	0.368	0.111
Height (cm)	154.74 ± 9.24	154.50 ± 6.45	2.595	0.921
Weight (kg)	45.77 ± 9.69	46.28 ± 8.56	0.073	0.852
BMI (kg/m^2^)	18.95 ± 3.38	19.38 ± 2.55	0.556	0.636

### 2.3. Procedures

With the assistance of trained research assistants and PE teachers, participants wore the accelerometers that had completed data initialization and parameter setting (e.g., subject name, start/stop date and time, biometric information) during regular PE classes. The device was firmly fastened around the participants’ left side of the waist by means of an elastic belt at the beginning of each PE class, because the waist is close to the centre of gravity of the body, the measurement of activities such as walking and running, is more accurate, and it is also comfortable for the subjects (71). The children were then asked to engage in their regular PE classes. When the class bell rang, the data collection began, and the researchers looked at whether participants wore the instrument throughout the PE class, the effective wearing time of the accelerometer was defined as 100% of the class time. Participants whose devices were removed in the middle of the test for personal or other reasons were recorded and their data were deleted. When the bell rang again, the data collection ended, and the accelerometers were removed at the end of each PE class. A hard drive connected to a computer was used to download activity counts stored by the accelerometers. The processing and analysis of the data was computed in ActiLife v 6.13.4 software.

### 2.4. PE classes

Both schools scheduled four 35 min PE classes per week in an outdoor sports field in accordance with the regulations of the Ministry of National Education. There is only one class per grade in each school, and the class in each school was led by a certified PE teacher, their teaching experience is 3 and 5 years respectively, and was assisted by one para-educator. A total of 48 sessions in six classes (fourth-grade: 16 sessions in both classes, fifth-grade: 16 sessions in both classes and sixth-grade: 16 sessions in both classes) were monitored over a 2 weeks period. The number of lessons selected aimed to provide coverage across different days, times, and activities, which aimed to reduce the potential bias that could arise from measuring MVPA in a limited number of instances or under specific conditions. Each PE class was recorded on a video recorder, and combined with the teachers’ lesson plans, we made a brief summary of the process and content of the PE classes for children with ID, as shown in Table 2. Each conventional PE class consists of three sections, namely the preparation section, the core section and the end section. The preparation section and the end section are basically the same for each grade, however in the core section, fourth graders played more group game play, while fifth and sixth graders had more skill practice, and teachers also gradually instilled PE knowledge in them.

**Table 2:** The basic process and content of PE class for children with ID.

Section	Activity	Duration (min)
Preparation		8
	Regular routines (e.g., taking attendance)	
	Warm-up (e.g., walking, jogging, jumping, and joint mobility)	
Core		20
	Teacher introduction of core exercise (group game play, e.g., traditional Chinese games; skill practice, e.g., ball sports)	
	Teacher’s demonstration	
	Teacher explanation of organizational arrangement	
	Students practice	
End		7
	Breathing exercises	
	Teachers’ summary	

### 2.5. Statistical analysis

All data analyses were conducted using IBM SPSS version 26.0 (IBM Corp., Armonk, NY, United States), and *p* value was set at 0.05. For data analysis, only the average time spent in MVPA during PE classes was considered. The MVPA % during the PE class was calculated by adding the MVPA minutes during 48 PE classes and dividing the sum by the minutes of total monitoring time during PE class, and the results were expressed as mean ± standard deviation (SD).

In the first step, the data distribution was analyzed with the Kolmogorov–Smirnov test. Since all variables conformed to the criteria for a normal distribution, further data treatment was performed using parametric tests.

In the second step, gender (boys and girls) and grade (fourth, fifth, and sixth grade) were the two independent variables in the statistical analysis; the dependent variable for this statistical analysis was the minutes of MVPA. The independent samples *t*-test was used to test the mean difference in MVPA between boys and girls, while analysis of variance (ANOVA) with multiple comparison tests (*post hoc*) per LSD was used to analyze the for mean difference in MVPA between grade-group.

In the next step, we conducted a Levene’s Test for Equality of Variances first, which yielded a significance value greater than 0.05, indicating the presence of homogeneity of variance; then, a 2 × 3 (gender × grade) two-way ANOVA was used to test mean differences in MVPA between boys and girls in the different grade groups. The LSD test was used as a *post hoc* test.

## 3. Results

**Figure 1:**
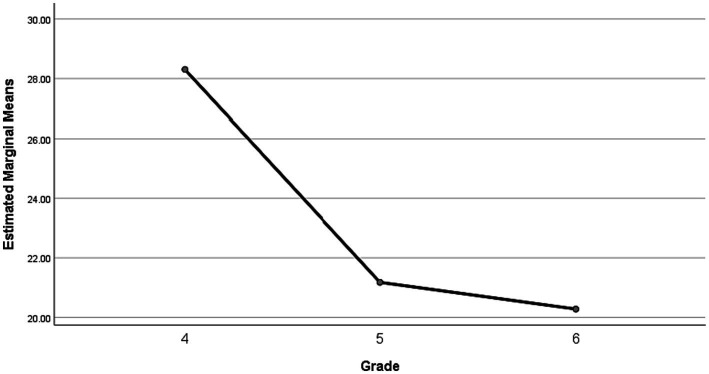
MVPA% during PE classes according to grade.

Table 3 showed the means, SD and percentual values of MVPA during school PE classes, according to grade and gender. For the entire sample, the mean time spent by children with ID on MVPA in PE class was 8.00 ± 2.10 min, which accounts for 22.88% of PE class time. The MVPA% (22.88%) during PE class, which is below the U.S. DHHS and the U.K. AfPE of recommendation of 50%. Boys spent significantly more time in MVPA during PE classes than girls (9.20 vs. 5.70 min, *p* < 0.05). As can be seen from the Figure 1, as the grade progresses, the mean time spent in MVPA during PE classes of children with ID shows a downward trend, as well as the mean time spent in MVPA during PE classes in fourth-grade was significantly higher than in fifth-grade and sixth-grade (9.15 vs. 7.61 vs. 7.25 min, *p* < 0.05), but no significant differences were found between fifth-grade and sixth grade (7.61 vs. 7.25 min, *p* < 0.05).

**Table 3:** MVPA during PE classes according to gender and grade group.

	Sample	Age (years)	MVPA (min)	MVPA/PE (%)
All sample	53	12.60 ± 1.66	8.00 ± 2.10	22.88 ± 6.01
Boys	35	12.34 ± 1.64	9.20 ± 1.47^*^	26.27 ± 4.19
Girls	18	13.11 ± 1.61	5.70 ± 0.83	16.28 ± 2.38
Grade 4	17	11.67 ± 1.28	9.15 ± 2.02^*^	26.13 ± 5.77
Grade 5	18	12.35 ± 1.73	7.61 ± 2.10	21.74 ± 6.00
Grade 6	18	13.78 ± 1.22	7.25 ± 1.77	20.71 ± 5.07

^*^Denotes significant difference (^*^p < 0.05); MVPA, moderate-to-vigorous intensity physical activity; PE, physical education.

**Figure 2:**
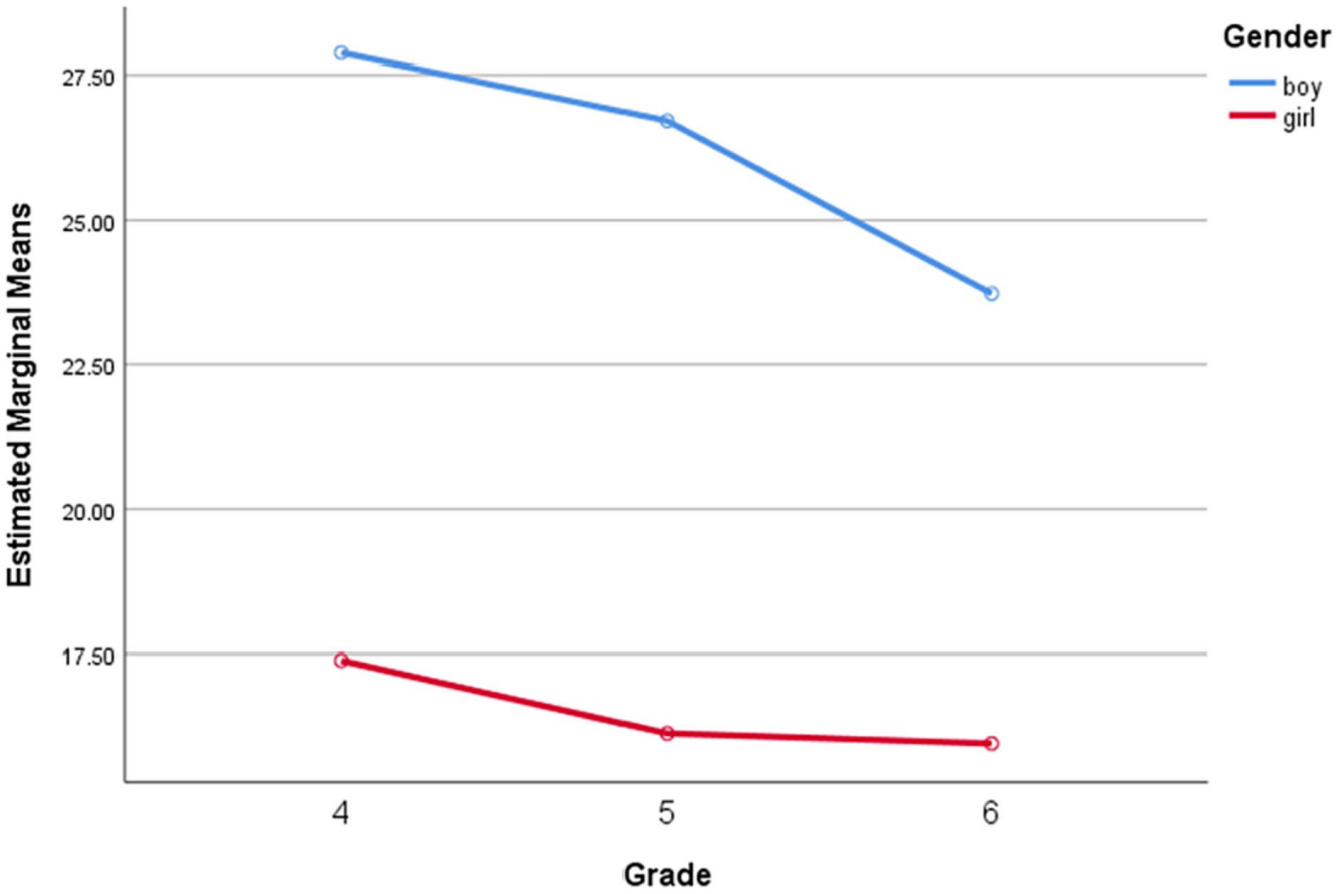
MVPA% of boys and girls with different grades during PE classes.

Table 4 showed the means, SD and percentual values of MVPA during PE classes of boys and girls with 3 different grades. Results revealed that the gender main effect was significant, *F*(1, 47) = 78.133, *p* < 0.001, *η*^2^ = 0.624; both grade and gender × grade interaction were not significant, *F*(2, 47) = 2.136, *p* = 0.129, *η*^2^ = 0.083, and *F*(2, 47) = 0.823, *p* = 0.443, *η*^2^ = 0.034, respectively. Figure 2 illustrated that boys spent significantly more time in MVPA during PE classes in each grade than girls, as well as boys and girls engage in MVPA gradually decreases as grade processes.

**Table 4:** MVPA of boys and girls with different grades during PE classes.

Grade	Sample	Boys’ MVPA		Girls’ MVPA	
		min	%	min	%
Grade 4	17	9.76 ± 1.60	27.88 ± 4.55	6.09 ± 0.31	17. 39 ± 0.90
Grade 5	18	9.35 ± 1.13	26.72 ± 3.24	5.68 ± 0.58	16.14 ± 1.64
Grade 6	18	8.31 ± 1.16	23.73 ± 3.32	5.59 ± 1.19	15.96 ± 3.40

MVPA, moderate-to-vigorous intensity physical activity.

## 4. Discussion

Given the beneficial effect of PE in promoting children’s PA levels, accurate identification and assessment of time spent in MVPA during PE class for children with ID can help increase the opportunities of them participating in MVPA – which is critical in development of appropriate PE class-based interventions. This present study is the first to objectively assess the MVPA participation of children with ID during PE class in special schools in mainland China. The results of our study indicate that children with ID failed to achieve the recommended 50% MVPA during PE class, and the amount of MVPA participation varied greatly between grade and gender. Specifically, according to grade, the time spent in MVPA during PE class decreases significantly with grade; and by gender, boys spent significantly more time in MVPA than girls, both in the entire sample and in each grade.

Consistent with previous studies (63, 64), the present study showed that children with ID did not meet the DHHS recommendation that students engage in MVPA for at least 50% of the time they spend during PE class. Only 22.88% of the time was spent in MVPA during PE class, but this proportion is significantly lower compared to the 49.6% reported in a study by Sit et al. (64). The first possible reason for this disparity is related to severity ratings of ID. The primary participants in their study were children with mild ID, while the primary participants in our study were children with severe ID. A previous review has showed that the severity ratings of ID is the strongest predictor of the PA levels of individuals with ID, higher severity of ID was related to decreased of PA levels (38). The second reason for this disparity may be attributed to differences in lesson content. In Sit’s study, participants were drawn from a school in Hong Kong with a strong emphasis on sports, where children frequently engaged in high-intensity PA during their PE classes. These activities often included sports such as basketball, soccer, and track and field events. Notably, Hong Kong schools are granted the flexibility to design a ‘school-based curriculum’ tailored to the unique educational needs of students with ID (64). In contrast, the schools in our study, situated in mainland China, predominantly offered traditional PA programs as part of their PE curriculum. These programs primarily featured activities like walking, jogging, and various ball games, with walking being the predominant form of exercise. This choice of activities reflects a more conservative and less intensive approach to PE for children with ID, which may inadvertently limit the intensity and variety of PA available to children with ID. Importantly, there are no specific guidelines or well-established special PE curriculum standards for children with ID in mainland China. Consequently, the PE curriculum for these students remains underdeveloped and lacks the customized and adaptive approach observed in Hong Kong. The third possible reason for this disparity is related to lesson length. The average lesson length in Sit’s study was 18.3 min, but the lesson length in our study was about twice as long as in their study. This directly leads to a significant percentage difference in MVPA during PE class due to shorter classes likely being more activity intensive. Finally, possibly the most important difference between the two studies were the different measurement methods of the PA. Direct observation tool-SOFIT was used to assess student PA during PE class in the Sit’s study. However, against accelerometry, validity correlations were low for SOFIT (67), which may overestimate the time students spend in MVPA during PE class (44). PA was measured objectively by accelerometer in our study. The accelerometer allows real time assessment of frequency, intensity and duration of PA (72, 73). This device has been used by a substantial number of authors because of its importance in the evaluation of PA patterns in a given context, allowing a critical comparison of results with other populations (74, 75). The proportion observed in our study closely aligns with the 25.81% reported in the study conducted by Pan et al. (63). This similarity could be attributed to a common factor shared between the studies, appears to be the content of the PE class. Curriculum content in both studies focused on light-to-moderate intensity activities. The similarity in the age range of participants is also noteworthy. Given the significant association between the PA levels of children with ID and their age (37), it becomes evident that various age groups could potentially exhibit distinct responses to identical curriculum content. Nevertheless, the participants in both studies fell within closely similar age ranges, this shared age factor suggests that the observed proportions might possess a particular relevance to the PA characteristics of this specific age cohort.

It was reported that even typically developing children also did not meet the recommended 50% of class time criterion, and the percentage of PE class time spent in MVPA was 44.8% on average (53). This shared pattern of not meeting the recommendation highlights a common challenge in promoting sufficient levels of MVPA in PE settings. This agrees with previous study (63) indicating that PA levels of children with ID were substantially lower during PE classes than those of typically developing children – which could be partially explained when we consider that children with ID are relatively physically inactive due to delayed motor development (76). Downs et al. (69) reported that children with ID showed transient intermittent activities, and the number of consecutive rounds decreased with the increase of intensity and duration. Moreover, various intertwined factors have contributed to the limited participation of children with ID in MVPA during PE classes. Neurologically, alterations in brain development and function contribute to reduced motor coordination and planning abilities. Impaired executive functions, linked to prefrontal cortex dysfunction, hinder their capacity to engage in structured activities (77). Sensory processing disparities, which are prevalent among individuals with ID, can significantly amplify discomfort during movement and consequently act as a deterrent to active PA participation (78). These disparities manifest in altered sensory perceptions and responses to stimuli from the environment. Children with ID may experience hypersensitivity or hyposensitivity to sensory inputs like touch, sound, or motion during PE classes, such as, the sensation of a ball hitting their body might trigger an overwhelming response, leading to discomfort or even pain. The fear of experiencing discomfort or sensory overload can lead to avoidance behavior, resulting in reduced PA involvement. From a physiological perspective, one of the factors contributing to the reduced PA levels observed in children with ID is hypotonia, it results from abnormalities in the neuromuscular system, leading to reduced muscle stiffness and decreased muscle resistance to passive movement (79). On the one hand, hypotonia translates to weakened muscle strength, which directly affects their capacity to initiate and sustain movements (80, 81). Muscles are responsible for generating force to perform various tasks, and when muscle tone is low, the force generated is insufficient, resulting in compromised movement efficiency. As a consequence, children with ID might struggle to maintain proper posture, execute coordinated movements, and perform tasks that require muscle strength, such as lifting objects. On the other hand, hypotonia often leads to early onset of fatigue (82). Due to weakened muscle fibers, the energy required to sustain physical activities is expended at a faster rate. This premature fatigue can significantly limit their endurance, making it challenging to engage in sustained activities or longer sessions of MVPA. While cognitive challenges can impede comprehension of instructions and rules (83). The absence of program adapted to their unique needs and capabilities not only curtails their participation but also hampers their potential to benefit fully from PE classes. Difficulties in peer interactions further impact engagement (84). Additionally, inadequate teacher training in adapting activities and individualized support further contributes to low activity levels (85).

Addressing these barriers necessitates a multifaceted approach. Tailored interventions encompassing adapted activities, fostering supportive environments, and empowering educators through training are essential. Creating inclusive and diverse PE programs that account for the unique needs of children with ID can effectively promote their active involvement. This, in turn, fosters improved levels of MVPA and unlocks the array of associated physical, cognitive, and social benefits. Encouragingly, existing research reinforces the potential to enhance the quality of PE class. Several studies have shown that programs designed to improve the quality of PE can actually increase the amount of time that students are engaged in MVPA to more than 50% of PE class time (54, 86, 87). Consequently, it is important to establish and implement high-quality PE in special education schools. In fact, some evidence suggests that training can improve teachers’ knowledge and confidence to successfully implement PA programming, which is also critically important to program success and children health (88, 89). Additionally, PE teachers could use positive reinforcement, praise, and rewards to motivate and encourage children with ID, such as using gestures, smiles, high-fives, or claps to visually reinforce positive behavior and effort. These strategies can help create a positive and supportive environment that enhances their participation, self-esteem, and enjoyment of PE classes.

Grade differences in MVPA during PE classes have been investigated using other types of instrumentation (e.g., SOFIT, heart rate telemetry) in previous studies (61, 90, 91), and generally older grade levels were more active than younger grade levels. In contrast, unlike the findings reported in typically developing children, the time spent in MVPA during PE class tended to decrease as children with ID moved grade in this study. For typically developing children, the increase in MVPA levels as grade levels rise can be linked to several factors. As children progress through school, they are exposed to a wider range of physical activities, sports, and organized games that promote active engagement. Additionally, they tend to develop better motor skills, coordination, and fitness levels with age and experience, which encourages greater participation in PA. The peer influence and effective teacher engagement, can also contribute to higher PA levels, as they engage in activities together and may be more motivated to participate as they grow older. However, for children with ID, considering that lesson length remained the same as the cohort moved from fourth grade to sixth grade, but children’s MVPA during PE classes decreased. The results of the current study could be influenced by growth, maturation, and environmental factors. The increase in age puts children with ID at risk of reduced PA (92, 93), younger children with ID are more active in facing various activities, while the older are quieter. The lack of adapted programming in schools, limited facilities, and insufficient teacher training in adapting activities for children with ID, can further contribute to the decline in MVPA levels among older children with ID. Interestingly, our analysis of the data revealed a noteworthy pattern: a significant decrease in the magnitude of variation in MVPA levels from fourth to fifth grade, followed by an absence of significant differences from fifth to sixth grade. This intriguing trend may be elucidated by examining the differences in teaching objectives and lesson content across these grade levels. Specifically, when comparing the fourth-grade to the older cohorts, it was evident that the fourth-grade curriculum placed a greater emphasis on game play activities while allocating comparatively less time to skill practice and the acquisition of PE knowledge. This distinction in curriculum focus could partially account for the observed shift in PA patterns. Previous study has demonstrated that students participating in team sports and game play activities tend to exhibit higher levels of MVPA compared to those engaging in other types of PE activities (94). The dynamic nature of team sports often involves continuous movement and competition, naturally promoting increased MVPA among participants. Conversely, the older grades, particularly fifth and sixth grades, may have seen a shift towards a more balanced curriculum, incorporating a wider range of PE activities, including skill development and theoretical knowledge. This broader curriculum approach, while beneficial for overall physical education, may have resulted in a stabilization of physical activity levels, leading to the insignificant differences observed from fifth to sixth grade. Whereas, in typically developing children, older grades are more likely to engage in activity continuously for longer periods of time, while younger grades tend to engage in activity intermittently (95). It is also possible that with the increase of grade, typically developing children have more choices than children with ID. They are often allowed to select a PE content of interest, rather than all students at a certain grade level participating in the same PE class, this promotes higher levels of student interest and participation (61). As highlighted in the study conducted by Sarradel et al. (91), there exists a demonstrable correlation between the content of PE classes and the varying PA levels exhibited by children, this sheds light on the nuanced relationship between the curriculum design within PE and the resultant PA engagement among students. These findings underscore the critical importance of incorporating diverse content options within PE curricula, as the choice of activities can yield varying PA outcomes among students. This highlights a significant implication for the development of future PE curricular programs, especially within the context of special education schools catering to children with ID. Specifically, it is essential for future PE curricula in special education schools to not only focus on the training of necessary physical and behavioral skills that children with ID require but also to include appropriate high-intensity activities. These high-intensity activities are crucial for achieving health-related levels of both the amount and intensity of PA. In other words, PE programs in special education schools should be designed to strike a balance between skill development and the provision of physically demanding activities. By doing so, these programs can not only address the specific needs of children with ID but also promote their overall physical well-being, ensuring that they engage in a sufficient and suitable level of PA to support their health and development.

The findings of this study support the notion that boys spent significantly more time engaged in MVPA than girls during PE classes, which is consistent with systematic review of Hollis et al. (52) on MVPA levels of typically developing children in elementary school. McKenzie et al. (96), Scruggs (97), and Kirkham-King et al. (95) assessed PA levels of children during elementary PE class with SOFIT, pedometer, and accelerometer respectively, and the results showed that boys are more active than girls. Whatever the measurement approach chosen to assess PA in PE class, it consistently shows wide variation between gender. Explanations for this variation may be primarily revolve around physiological and psychosocial factors (98). Specifically, a previous study revealed that motives for participating in a variety of PA differed between gender groups, with male students rating fun and learning new skills higher and being more likely to engage in vigorous activity, whereas female participants preferred more sedate activities (99). Girls tend to be habitually less active than boys and their levels of activity participation start to decline at an earlier age (100). In addition, as children get older, gender disparities gradually increase, girls may encounter a series of drastic changes in body shape, function, endocrine and physical fitness, these are seen as barriers to participation in PA, thus affecting girls’ enthusiasm to participate in PA (101, 102). The potential public health impact of low levels of PA for girls can be severe (103). Based on these findings, it could be suggested that future targeted interventions should be considered, for example, increase the content of PE class that girls are interested in (e.g., dance and fitness). For example, a study indicated that girls participated in higher MVPA during fitness and dance than boys (91). Additionally, where possible, consider increasing the time girls spend in MVPA during PE class through a form of separate teaching (104).

Several potential limitations of this study should be acknowledged. First, the cross-sectional nature of this study and the data collected over a short period time may affect the causal inference that was made. Second, a limited number of participants were recruited from one special school located in one city in China, this lack of representativeness could potentially limit the generalizability of our findings to a broader spectrum of children with ID. This highlights that future studies should consider a longitudinal approach involving a large number of children with ID from different school settings and regions. Third, participants were aware that their PA levels were being monitored by the devices and the researchers, which may have encouraged more PA than usual, this might not accurately reflect their PA levels during daily PE classes, hence might slightly affect the ecological validity. However, the PE teachers made every effort to encourage participants to continue their regular PA patterns during the monitoring period to avoid this potential bias. Finally, since motor development was positively correlated with PA of children with ID (37), disparities in this aspect may affect MVPA levels. Last, the absence of a comparative group consisting of typically developing controls hinders the comprehensive exploration of disparities and resemblances in MVPA during PE classes between children with ID and their typically developing peers. Future studies that incorporate both these cohorts would offer essential and novel insights into PA levels among Chinese mainland children with ID.

Meanwhile, a notable strength of this study lies in its utilization of accelerometry to objectively assess PA during PE classes. Additionally, it is the first multilevel analysis (grades level, gender) of changes in MVPA during PE classes for children with ID, a type of analysis that has been underutilized in this special population. This information is important because the results empower schools and PE educators to develop and increase multilevel PA interventions and PE programs among children with ID.

## 5. Conclusion

Findings from this study indicate that the proportion of PE class spent in the MVPA of children with ID was significantly lower than the recommended 50% value. Also, the amount of MVPA participation varied by the grade and gender. Considering that PE may make an important contribution to the participation of children with ID in regular PA, the formulation and optimization of curriculum content cannot be ignored, and the quantity and quality of PE classes need to be improved and strengthened to meet the needs of children with intellectual disabilities, while increasing their physical competence. Thus, in order to help children with ID achieve MVPA goals, it is suggested that (1) strive for multiple times PE classes per week to promote both variety and regularity to ensure consistent engagement in PA for children with ID. (2) Encourage activities that elevate heart rate and cause moderate sweating while considering the individual’s capabilities. Implement activities that engage children with ID, while also monitoring their comfort. (3) Each PE class should ideally consist of at least 30 min of MVPA, gradually increase the duration of activities as the children with ID’s fitness levels improve, ensuring safety and avoiding exhaustion. (4) Provide a variety of activities that cater to different interests and abilities between boys and girls, as well as different grade, ensuring that every child with ID can actively participate. And incorporate both structured and unstructured activities, such as team sports, dance, recreational games, and functional exercises.

Future research should concentrate on understanding motivations of children with ID for participating in PA, which could have important implications for the content of PE classes and the extracurricular activities offered by special education schools.

## Data availability statement

The raw data supporting the conclusions of this article will be made available by the authors, without undue reservation.

## Ethics statement

The study involving humans was approved by the Universiti Malaya 163 Research Ethics Committee (UMREC) (UM.TNC2/UMREC_1590). The studies were conducted in accordance with the local legislation and institutional requirements. Written informed consent for participation in this study was provided by the participants’ legal guardians/next of kin.

## Author contributions

YH and RR: conceptualization, methodology, formal analysis, writing – original draft preparation, writing – review and editing, and visualization. RR: supervision. All authors contributed to the article and approved the submitted version.
